# Duties of the Hour

**Published:** 1893-09

**Authors:** 


### DUTIES OF THE HOUR.

The conception of human welfare is not a fixed, but an ever-growing one, imposing new and increasing duties upon us. Every advance in intelligence brings with it not only an increase in comfort and happiness, but new obligations to all who partake of it. Consider, first, the duties of labor and rest; one is quite as important as the other. A well-known editor in retiring recently from his active labors took no little pride in the fact that he had worked continuously for forty years without taking a vacation. While his work has been able and useful, we doubt if his pride in his record of continuous work is well grounded, but believe it to be the exception that proves the rule. He had done well without vacation, but what new light might have shone from his journal had he stopped writing long enough, at least once a year, to have renewed his physical and mental vigor and gained new inspirations, which would have given greater variety and breadth to his work. And thus it is with us, rest, diversion, and recreation are necessary if we would give our clients and the profession our best efforts.

Viewing it again from the stand-point of one's health, the care of the health is becoming more and more insisted upon, not only as a duty to ourselves but to those dependent upon us. We are constantly confronted with physical and mental break-downs, due to too close application to work, and too little attention to personal health. Recently, in connection with the World's Columbian Exposition, several have been recorded. The victims have failed in nerve-strength, and practically laid down their lives by too close application to their vocation,—one of them a noble woman, who, from the severe strain of manifold duties and zealous devotion to a noble work,—the speech-teaching of deaf children,—sacrificed her life. While we should show the utmost fidelity to our profession, we should at the same time heed these warnings, and not destroy or overtax our physical and mental strength. The writer now calls to mind several members of the dental profession who, after laboring long hours at the chair, confine themselves far into the night, working upon some mechanical device which may or may not have a bearing upon their professional life; or endeavoring to solve the phenomena of spiritualism, etc. We hear them spoken of as growing eccentric, but insanity, physical collapse, or untimely death will be the penalty.

Consider, now, for a moment, the duty of justice to each other; this is gradually embracing a wider area. It is upon this one word, justice, that our entire ethical code is based. And we cannot properly deal with ethical or moral questions until we free ourselves from all personal piques, resentments, and preferences; can appreciate the positions of others, and are ready to put ourselves in the places of those with whom we may be associated, and choose their welfare as well as our own.

Again, we have a certain duty we should discharge towards the *growth* of our profession. How many read the best journals? With each recurring month our journals bring before us the thoughts and methods of those who are studiously and steadily advancing; and the dentist who has "no time to read" and learn, and thus add to his own usefulness and help to exalt his profession in public esteem, will surely fall behind. We must be progressive; one dare not in these times attempt to stand still, for, as another has aptly said, "A state of rest means rust, and rust means retrogression." How many are, through the journals or societies, contributing to its development according to the breadth of their intelligence? Intellectual progress is now greater than it ever has been in the history of the world. There are, too, more bright young men and women entering the professions than ever before, and it should be remembered that every one is worth just as much to his profession and to the world as he gives it.

We, therefore, with our growing intelligence, with the new inspirations we have received from our vacations,—our time of rest, relaxation, and meditation,—should return to our posts of duty prepared not only to hold to the conventional and long-established obligations, but to welcome all messages which tend to arouse us from our lethargy, and point out our weaknesses and paths of duty.

Most of us who think at all have moments when we catch glimpses of something higher and better than we have yet embraced. Let us see to it that we do not discard these suggestions because they may at the time be unpopular. Professor Fowler, of Oxford, England, says truly, " We may not unreasonably hope that there will be a stricter sense of justice, a more complete realization of duty, more delicacy of feeling, a greater refinement of manners, more kindliness, quicker and wider sympathies in the coming generations than there is among ourselves. . . . In all departments of human activity we are bound to do for our successors what our predecessors were bound to do, and mostly did, for us,—transmit the heritage we have received, with all the additions and adaptations which the new experiences and changed conditions of life have rendered necessary or desirable."

